# Prevalence and factors associated with health insurance coverage in urban sub-Saharan Africa: Multilevel analyses of demographic and health survey data

**DOI:** 10.1371/journal.pone.0264162

**Published:** 2022-03-04

**Authors:** Hubert Amu, Kwamena Sekyi Dickson, Kenneth Setorwu Adde, Kwaku Kissah-Korsah, Eugene Kofuor Maafo Darteh, Akwasi Kumi-Kyereme

**Affiliations:** 1 Department of Population and Behavioural Sciences, School of Public Health, University of Health and Allied Sciences, Hohoe, Ghana; 2 Department of Population and Health, University of Cape Coast, Cape Coast, Ghana; Himachal Pradesh Medical College: Indira Gandhi Medical College, INDIA

## Abstract

**Introduction:**

With the vision of achieving Universal Health Coverage (UHC) by the year 2030, many sub-Saharan African (SSA) countries have implemented health insurance schemes that seek to improve access to healthcare for their populace. In this study, we examined the prevalence and factors associated with health insurance coverage in urban sub-Saharan Africa (SSA).

**Materials and methods:**

We used the most recent Demographic and Health Survey (DHS) data from 23 countries in SSA. We included 120,037 women and 54,254 men residing in urban centres in our analyses which were carried out using both bivariable and multivariable analyses.

**Results:**

We found that the overall prevalence of health insurance coverage was 10.6% among females and 14% among males. The probability of being covered by health insurance increased by level of education. Men and women with higher education, for instance, had 7.61 times (95%CI = 6.50–8.90) and 7.44 times (95%CI = 6.77–8.17) higher odds of being covered by health insurance than those with no formal education. Males and females who read newspaper or magazine (Males: AOR = 1.47, 95%CI = 1.37–1.57; Females: AOR = 2.19, 95%CI = 1.31–3.66) listened to radio (Males: AOR = 1.29, 95%CI = 1.18–1.41; Females: AOR = 1.42, 95%CI = 1.35–1.51), and who watched television (Males: AOR = 1.80, 95%CI = 1.64–1.97; Females: AOR = 1.86, 95%CI = 1.75–1.99) at least once a week had higher odds of being covered by health insurance.

**Conclusion:**

The coverage of health insurance in SSA is generally low among urban dwellers. This has negative implications for the achievement of universal health coverage by the year 2030. We recommend increased public education on the benefits of being covered by health insurance using the mass media which we found to be an important factor associated with health insurance coverage. The focus of such mass media education could target the less educated urban dwellers, males in the lowest wealth quintile, and young adults (15–29 years).

## Introduction

Global discussions on health policy have over the past three decades been dominated by universal health coverage (UHC) and the protection of people from the burden of catastrophic out-of-pocket payments for healthcare [1–5]. The World Health Organisation (WHO) [6] observed that the high cost of out-of-pocket health expenditure is a major barrier to the achievement of UHC which is a target of the sustainable development goals (SDG). Other studies have shown that health insurance coverage is a key factor in accelerating progress towards the achievement of UHC [7–9].

In pursuit of UHC, sub-Saharan African (SSA) countries such as Ghana, Kenya, Mali, Nigeria, Rwanda, South Africa, Tanzania, and Zimbabwe have implemented health insurance schemes that seek to improve access to healthcare for their populace [9–11]. Access to healthcare through health insurance, however, remains limited [12, 13]. With this premise, Amu, Dickson, Kumi-Kyereme, and Darteh [14] examined the variations in health insurance coverage in four countries (Ghana, Kenya, Nigeria, and Tanzania) which were the first SSA countries to launch developmental plans in the early 1960s. Amu et al. [14] realised that in most of the countries, the probability of being covered by health insurance was lower among urban dwellers. Country-level studies conducted in Kenya [15], Kyrgyzstan [16], Bangladesh [17], and Nepal [18] have also reported lower health insurance coverage among urban dwellers.

The WHO [19] also argues that more than 90% of people living in slum conditions are located in urban areas which invariably results in health inequalities. These health inequalities are circumstances in which people grow, live, work, and age among others. This is also a result of urban planners lacking basic information on urban dwellers. Hence, proportions of urban dwellers remain in the dark and their health challenges are usually overlooked by governments and decision-makers [19]. Our objective was, therefore, to examine the prevalence and factors associated with health insurance coverage among urban dwellers in 23 SSA countries. We used DHS data for our analysis due to the nationally representative nature of the surveys in the respective countries. The findings will provide a broader perspective on health insurance coverage in SSA. Additionally, the study will enable policymakers to have a better understanding of health insurance coverage among the urban population in SSA and also proffer suggestions for improving the status quo.

## Materials and methods

### Study design

The study used data from the Demographic and Health Surveys (DHS) were collected in 23 countries across SSAs. The DHS conducts nationally representative surveys in over 85 low- and middle-income countries between 2010 and 2019 around the world using a recurrent cross-sectional research design. The surveys concentrate on maternal and child health, physical activity, sexually transmitted infections, fertility, health insurance, tobacco use, and alcohol consumption. They mainly provide data to monitor the demographic and health profiles of the respective countries [20]. Our study, however, focused on those aged 15–64 as coverage of health insurance has implications for maternal and overall adult health.

### Data collection procedure

The surveys’ data collection technique includes using a standard questionnaire that is equivalent across nations to collect information from women aged 15–49 and men aged 15–59, as well as information on their children. The questionnaire is frequently translated into the major local languages of the participating countries. The DHS claims that the translated questionnaires, along with the English-language version, are pretested in English and the local dialect to guarantee their validity. After that, the pre-test field workers engaged in a lively discussion of the questions, making suggestions to improve all versions. Following field practice, a debriefing session with the pre-test field personnel is held, and the questionnaires are modified depending on the lessons learned. Details on the sampling methodology, procedures, and implementation can be found elsewhere [21].

### Sampling procedure and size

The sampling procedure employed in the surveys involves a two-stage stratified sampling procedure, where countries are grouped into urban and rural areas. The first stage involves the selection of clusters usually called enumeration areas (EAs) and the second stage consists of the selection of a household for the survey. The study by Aliaga and Ruilin [21] provides details of the sampling process. For this study, only women and men residing in urban centres were included in our analyses. A total of 54,254 men and 120,037 women who had complete information on all the variables of interest were included in the study (Table 1). We relied on the Strengthening the Reporting of Observational Studies in Epidemiology (STROBE) statement in writing the manuscript [22]. The dataset is freely available for download at https://dhsprogram.com/data/available-datasets.cfm (accessed on 17th February 2021)

**Table 1:** Background characteristics and coverage of health insurance in SSA.

Variables	Males		Females	
	Frequency (N = 54,254)	Proportion covered by health insurance	Frequency (N = 120,037)	Proportion covered by health insurance
**Age**				
15–19	10,527	8.2	25,676	7.2
20–24	8,974	9.8	22,933	8.4
25–29	8,292	13.4	22,114	10.5
30–34	7,289	16.2	17,401	12.7
35–39	6,239	16.9	14,362	13.3
40–44	5,026	19.2	9,788	14.9
45–49	3,774	20.0	7,763	13.2
50–54	2,450	20.4	-	-
55–59	1,446	19.0	-	-
60–64	237	14.5	-	-
**Wealth status**				
Poorest	1,666	3.1	3,783	3.7
Poorer	2,407	6.0	5,256	4.5
Middle	5,718	6.8	12,817	6.0
Richer	14,836	10.5	32,774	7.0
Richest	29,627	18.5	65,407	14.2
**Level of education**				
No education	5,206	3.1	20,302	3.4
Primary	11,198	7.1	28,671	5.0
Secondary	27,979	13.2	57,153	12.0
Higher	9,871	30.0	13,911	26.6
**Marital status**				
Never in union	25,021	10.2	42,703	10.0
Married	22,543	19.0	52,945	12.5
Cohabitation	4,626	11.1	13,170	7.6
Widowed	314	14.3	3,153	8.6
Divorced	617	20.0	2,682	8.7
Separated	1,133	9.6	5,384	7.2
**Frequency of reading newspaper or magazine**				
Not at all	27,222	8.8	80,431	7.5
Less than once a week	11,244	11.9	21,362	12.9
At least once a week	15,788	24.6	18,244	21.4
**Frequency of listening to radio**				
Not at all	9,199	8.5	35,209	6.3
Less than once a week	10,847	9.8	29,464	10.0
At least once a week	34,208	16.9	55,364	23.1
**Frequency of watching television**				
Not at all	11,212	6.3	34,594	4.3
Less than once a week	10,255	8.6	21,793	9.2
At least once a week	32,787	18.4	63,650	25.5

‘-’ indicate no values.

### Study variables

The outcome variable of this study was health insurance coverage. This was derived from the question “are you covered with any health insurance?”. Response is coded as 0 = “No” and 1 = “Yes”. The explanatory variables were age, wealth status, level of education, marital status, frequency of reading newspaper or magazine, frequency of listening to the radio, and frequency of watching television. Age was recoded as 15–19, 20–24, 25–29, 30–34, 35–39, 40–44, 45–49, 50–54, 55–59, 60–64. Wealth status was categorized as poorest, poorer, middle, richer, and richest. Education was classified into four categories: no education, primary education, secondary education, and higher education. The frequency of reading newspaper or magazine, frequency of listening to radio, and frequency of watching television were respectively captured as not at all, less than once a week, at least once a week, and almost every day. Our study variables and codings were based on previous literature [12, 14, 15] and their availability in the DHS dataset of selected SSA countries.

### Statistical analysis

We employed both descriptive and inferential analytical approaches. First, we computed the proportion of males and females who were covered by health insurance (see Table 1). Following the hierarchical nature of the data set, a multilevel logistic regression model was employed. This comprises fixed effects and random effects [23]. The fixed effects of the model were gauged with binary logistic regression which resulted in odds ratios (ORs) and adjusted odds ratios (AORs) (see Tables 2 & 3). The random effects on the other hand were assessed with Intra-Cluster Correlation (ICC) [24] (see Tables 2 & 3). Regarding the model building process, Model 1 is an empty model that looked at the ICC. Model 2 looks at the individual variables. It looks at the effects of the individual variables on the empty model. Model 3 looks at the effects of the Household variables on the empty model. Model 4 is the complete model that combined both the individual and the household variables. The complete model looks at the relationship of the explanatory variables (individual and household) on the outcome variables.

**Table 2:** Multilevel binary logistic regression results on the factors associated with health insurance coverage among males in SSA.

Variables	Model 1	Model 2 OR (95% CI)	Model 3 OR (95% CI)	Model 4 AOR (95% CI)
Age				
15–19	-	1	-	1
20–24	-	0.81***(0.73, 0.90)	-	0.82***(0.73, 0.91)
25–29	-	0.99(0.88, 1.11)	-	0.99(0.88, 1.11)
30–34	-	1.19**(1.05, 1.35)	-	1.18*(1.04, 1.34)
35–39	-	1.32***(1.16, 1.51)	-	1.31***(1.14, 1.49)
40–44	-	1.57***(1.36, 1.80)	-	1.53***(1.33, 1.76)
45–49	-	1.71***(1.48, 1.97)	-	1.67***(1.45, 1.94)
50–54	-	1.80***(1.54, 2.10)	-	1.76***(1.51, 2.07)
55–59	-	1.71***(1.42, 2.07)	-	1.67***(1.38, 2.01)
60–64		1.38(0.91, 2.11)	-	1.37(0.89, 2.09)
***Level of education***				
No education	-	1	-	1
Primary	-	1.88***(1.61, 2.21)	-	1.84***(1.57, 2.16)
Secondary	-	3.87***(3.34, 4.49)	-	3.67***(3.16, 4.26)
Higher	-	8.54***(7.31, 9.97)	-	7.61***(6.50, 8.90)
***Marital status***				
Never in union	--	1	-	1
Married	-	1.66***(1.52, 1.82)	-	1.69***(1.55, 1.85)
Cohabitation	-	1.12(0.99, 1.11)	-	1.15*(1.02, 1.31)
Widowed	-	1.13(0.78, 1.63)	-	1.15(0.80, 1.66)
Divorced	-	1.52**(1.19, 1.95)	-	1.54**(1.20, 1.98)
Separated	-	0.79*(0.63, 0.99)	-	0.81(0.64, 1.01)
***Frequency of reading newspaper or magazine***				
Not at all	-	1	-	1
Less than once a week	-	0.91*(0.85, 0.99)	-	0.90**(0.83, 0.97)
At least once a week	-	1.49***(1.40, 1.60)	-	1.47***(1.37, 1.57)
***Frequency of listening to radio***			-	
Not at all	-	1	-	1
Less than once a week	-	1.18**(1.06, 1.31)	-	1.18**(1.06, 1.33)
At least once a week	-	1.29***(1.18, 1.41)	-	1.29***(1.18, 1.41)
***Frequency of watching television***			-	
Not at all	-	1		1
Less than once a week	-	1.26***(1.13, 1.40)	-	1.19**(1.07, 1.31)
At least once a week	-	2.01***(1.84, 2.19)	-	1.80***(1.64, 1.97)
***Wealth status***	-	-		
Poorest	-	-	1	1
Poorer	-	-	1.63***(1.28, 2.08)	1.28(0.99, 1.65)
Middle	-	-	1.63***(1.31, 2.03)	1.11(0.88, 1.39)
Richer	-	-	2.37***(1.93, 2.91)	1.24(0.99, 1.53)
Richest	-	-	4.70***(3.85, 5.75)	1.69***(1.37, 2.08)
**Random effect result**	-	-		-
PSU variance (95% CI)	0.52(0.45, 0.62)	0.51(0.42, 0.60)	0.54(0.46, 0.64)	-
ICC	14%	13%	14%	13%
LR Test	Chi square = 1010.13 P valve = 0.0000	Chi square = 861.57 P valve = 0.0000	Chi square = 1001.89 P valve = 0.0000	Chi square = 870.71 P valve = 0.0000
Wald chi-square		4091.23	941.56	4161.15
Model fitness				
Log-likelihood	-20926.26	-18535.01	-20396.23	-18474.46
AIC	41856.51	37120.01	40804.45	37006.91
N	54,254	54,254	54,254	54,254

*p<0.05

**p<0.01

*** p<0.001.

**Table 3:** Multilevel binary logistic regression results on the factors associated with health insurance coverage among females in SSA.

Variables	Model 1	Model 2 OR (95% CI)	Model 3 OR (95% CI)	Model 4 AOR (95% CI)
Age				
15–19	-	1	-	1
20–24	-	0.92*(0.86, 0.99)	-	0.93(0.86, 1.00)
25–29	-	1.25***(1.16, 1.35)	-	1.26***(1.17, 1.37)
30–34	-	1.77***(1.63, 1.93)	-	1.78***(1.64, 1.93)
35–39	-	1.99***(1.83, 2.18)	-	2.00***(1.84, 2.19)
40–44	-	2.51***(2.29, 2.75)	-	2.50***(2.28, 2.75)
45–49	-	2.39***(2.16, 2.66)	-	2.38***(2.15, 2.64)
***Level of education***				
No education	-	1		1
Primary	-	1.58***(1.44, 1.73)	--	1.57***(1.43, 1.71)
Secondary	-	3.85***(3.55, 4.18)		3.81***(3.51, 4.14)
Higher	-	7.87***(7.18, 8.64)	--	7.44***(6.77, 8.17)
***Marital status***	-		-	
Never in union		1		1
Married	-	1.38***(1.30, 1.47)	-	1.38***(1.30, 1.46)
Cohabitation	-	0.88**(0.81, 0.95)	-	0.88**(0.81, 0.96)
Widowed	-	0.91(0.79, 1.05)	-	0.92(0.80, 1.06)
Divorced	-	0.73***(0.63, 0.85)	-	0.74***(0.63, 0.86)
Separated		0.68***(0.60, 0.77)		0.68***(0.60, 0.77)
***Frequency of reading newspaper or magazine***				
Not at all	-	1		1
Less than once a week	--	1.04(1.20, 1.36)		1.03(0.98, 1.09)
At least once a week	-	1.40***(1.32, 1.47)	-	1.39***(1.31, 1.46)
***Frequency of listening to radio***				
Not at all	-	1	-	1
Less than once a week	-	1.28***(1.20, 1.36)	-	1.27***(1.20, 1.36)
At least once a week	-	1.42***(1.35, 1.51)	-	1.42***(1.35, 1.51)
***Frequency of watching television***				
Not at all	-	1		1
Less than once a week	-	1.59***(1.48, 1.71)	-	1.56***(1.45, 1.68)
At least once a week	-	1.96***(1.85, 2.08)	--	1.86***(1.75, 1.99)
***Wealth status***				
Poorest	-	-	1	1
Poorer	-	-	1.21*(1.03, 1.45)	0.87(0.73, 1.04)
Middle	-	-	1.46***(1.26, 1.69)	0.85*(0.73, 0.99)
Richer	-	-	1.49***(1.30, 1.71)	0.65***(0.56, 0.75)
Richest			3.29***(2.88, 3.76)	0.96(0.82, 1.10)
**Random effect result**				
PSU variance (95% CI)	0.85 (0.74, 0.97)	0.99(0.87, 1.14)	0.81(0.71, 0.93)	0.98(0.86, 1.12)
ICC	20%	23%	19%	22%
LR Test	Chi square = 3162.84 P valve = 0.0000	Chi square = 3287.63 P valve = 0.0000	Chi square = 3024.89 P valve = 0.0000	Chi square = 3245.72 P valve = 0.0000
Wald chi-square		6997.4	1530.1	7156.5
Model fitness				
Log-likelihood	-38620.8	-34552.9	-37794.8	-34451.2
AIC	77245.67	69155.83	75601.63	68960.31
N	120,037	120,037	120,037	120,037

*p<0.05

**p<0.01

*** p<0.001.

The sample weight (v005/1,000,000) was applied in all the analyses to control for over and under-sampling. All the analyses were carried out using STATA version 14.2. We assess the fitness of the models with the Likelihood Ratio (LR) test. The presence of multicollinearity between the independent variables was checked before fitting the models. The variance inflation factor (VIF) test revealed the absence of high multicollinearity between the variables (Mean VIF = 2.67 for males and, mean VIF = 2.27 for females).

## Results

### Coverage of health insurance in urban SSA

**Fig 1:**
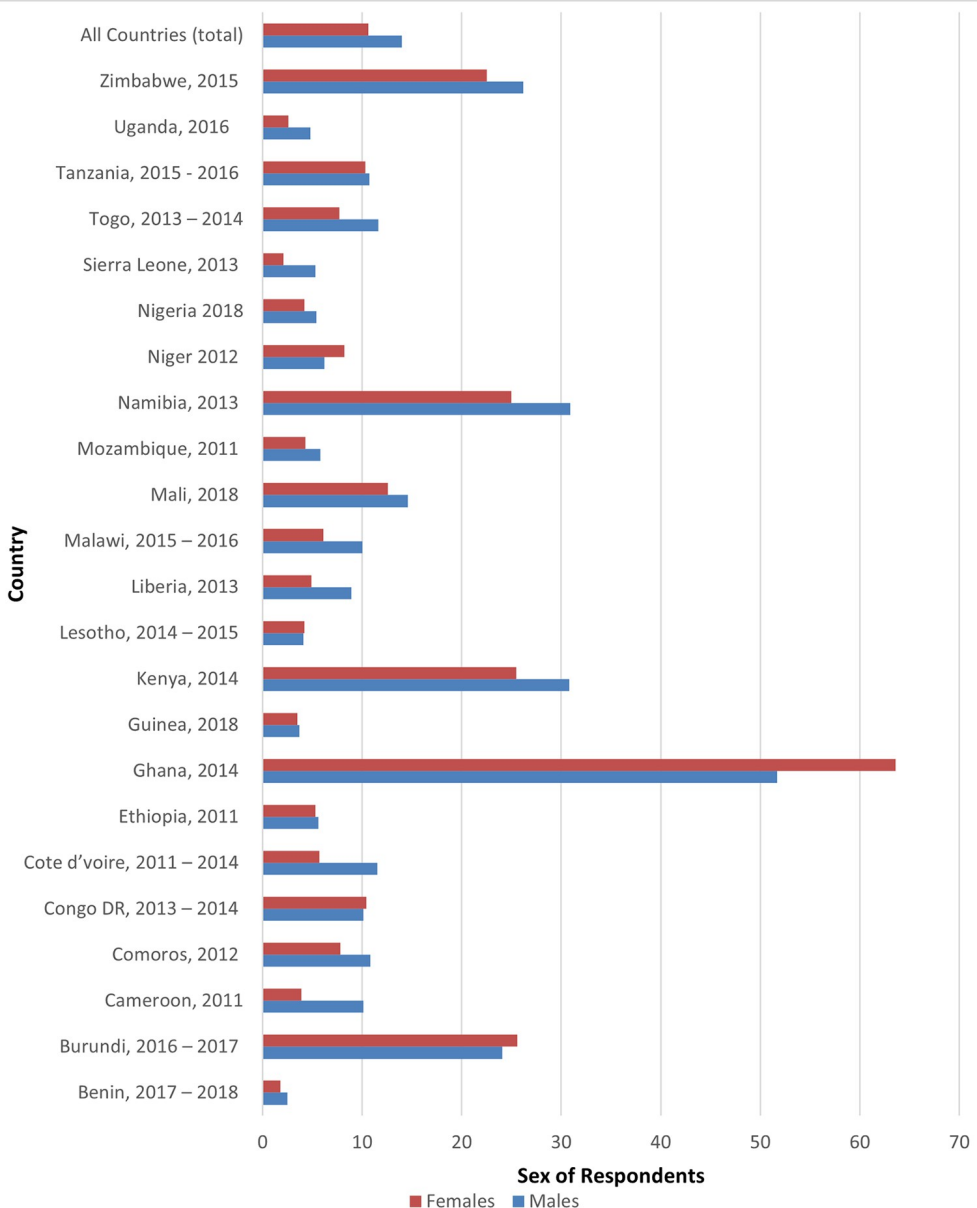
Coverage of health insurance in urban SSA.

Fig 1 presents coverage of health insurance among males and females in the 23 countries included in our analysis. The overall prevalence of health insurance coverage was 10.6% among females and 14% among males. The highest percentage coverage was recorded in Ghana (females = 63.6%, males = 51.7%) while the lowest was recorded in Benin (females = 1.8%, males = 2.5%).

Table 1 presents the coverage of health insurance by background characteristics dichotomized by sex. Among males, the highest proportion of coverage was recorded by those in their early 50s (20.4%). The highest proportionate coverage by age among females was, however, by those in their early 40s (14.9%). We found that the proportion of health insurance coverage increased by wealth status and level of formal education respectively among males and females. In terms of marital status, while divorced respondents had the highest coverage among males (20%), it was those who were married that had the highest prevalence among females (12.5%). Concerning mass media exposure and health insurance coverage, we found that among males, the highest proportions were recorded among those who read newspapers or magazines (24.6%), listened to radio (16.9%), and watched television (18.3%) at least once every week, respectively. Among females, it was those who read newspaper or magazine almost every day (24.1%), listened to radio at least once a week (13.3%), and watched television at least once a week (154.6%)

### Factors associated with health insurance coverage in SSA

We present multilevel logistic regression analyses on the factors associated with health insurance coverage. The main results presented are the multivariable model (Model 4) for males (Table 2) and females (Table 3). Age, wealth status, education, marital status, frequency of reading newspaper or magazine, frequency of listening to radio, and frequency of watching television were significantly associated with the coverage of health insurance among males and females. Regarding age, we found that among men, respondents who were 60–64 years old (AOR = 1.37, 95%CI = 0.89, 2.09) had higher odds of being covered by health insurance compared with those 15–19 years old. Among females, those in their last reproductive years (45–49) had 2.38 times higher odds (95%CI = 2.15, 2.64). For both males and females, the probability of being covered by health insurance increased by level of education. Men and women with higher education had 7.61 times (95%CI = 6.50, 8.90) and 7.44 times (95%CI = 6.77, 8.17) higher odds to be covered by health insurance than those with no formal education. Respondents who were married also recorded the highest probabilities of being covered by health insurance among males (AOR = 1.69, 95%CI = 1.55, 1.85) and females (AOR = 1.38, 95%CI = 1.30, 1.46) respectively.

Concerning the effects of mass media exposure on health insurance coverage, we found that males and females who read newspaper or magazine (Males: AOR = 1.47, 95%CI = 1.37, 1.57; Females: AOR = 2.19, 95%CI = 1.31, 3.66) listened to radio (Males: AOR = 1.29, 95%CI = 1.18, 1.41; Females: AOR = 1.42, 95%CI = 1.35, 1.51), and who watched television (Males: AOR = 1.80, 95%CI = 1.64, 1.97; Females: AOR = 1.86, 95%CI = 1.75, 1.99) at least once a week had higher odds of being covered by health insurance than men who did not. For wealth status, we found that the richest men (AOR = 1.69, 95%CI = 1.37, 2.08) were those who recorded the highest probability of being covered by health insurance. Among women, our bivariable analysis showed that the odds of being covered by health insurance increased with wealth status. This effect, however, changed in the multivariable analysis where women in all other wealth quintiles (poorer, middle, richer, and richest) were respectively less likely to be covered by health insurance compared with the poorest.

## Discussion

In this study, we examined health insurance coverage among males and females in urban centres of 23 SSA countries. We found that the overall prevalence of health insurance coverage was 11% among females and 14% among males. There were, however, variations in country-level prevalence with the highest proportion being recorded in Ghana and the lowest in Benin. The less than 15% coverage recorded in our study is worrying as it has negative implications regarding the achievement of universal health coverage of at least 80% which SSA countries, as well as other developing regions of the world, have committed to achieving as part of the global sustainable development goals (SDGs).

The low coverage recorded in most of the countries could be attributed to the myriad of challenges that bedevil the respective insurance schemes of these SSA countries. In Benin, for instance, low capital investments, implementation challenges, and the fragmented nature of existing health insurance policies make subscription to the schemes unattractive to indigenes. The many challenges have even threatened the implementation of mandatory social insurance for health called Assurance pour le Reinforcement du Capital Humain (ARCH) [25]. In Kenya, high subscription cost, insurance fraud, and lack of knowledge of the populace on health insurance, premium undercutting, poor underwriting, and negative perception of the populace towards health insurance have been the factors hindering high coverage of health insurance [26–30].

The less than 10% coverage we recorded in Nigeria could also be attributed to the systemic challenges that have saddled social health insurance in the West African country for decades. These included a lack of financial protection by health insurance from catastrophic out-of-pocket payments by clients, lack of medical infrastructure and equipment, inequitable allocation of resources for the provision of health insurance, corruption, low state spending on health, poor quality of services provided, fragmentation of pooled funds, and inability to extend health insurance coverage to the poor, vulnerable, and the informal sector (PVIS) [31–37].

In our study, the probability of being covered by health insurance increased with the level of education. Males and females with the highest levels of formal education, for instance, had the highest odds of being covered by health insurance. In a sub-region where formal education is now gradually gaining grounds especially for women [38, 39], this finding points to the important role that formal education plays in positively informing the health decisions of women as also realised in previous studies in SSA [40–47]. We found that the proportion and the probability of being covered by health insurance among males and females were respectively highest for those who were married. This finding could have been informed by the fact that couples after getting married are more likely to start a family and, therefore may need to get insurance coverage for unforeseen health challenges including sickness of a spouse, as well as reduce household out-of-pocket payments on antenatal, delivery, and postnatal care [48].

We found that among women, the probability of being covered by health insurance declined with increasing wealth status. This finding corroborates the observations made by other studies [14, 49, 50] and implies that among women, health insurance policies are meeting the pro-poor frameworks upon which they were designed. The poor in society do not have the necessary financial strength to afford the cost of accessing health out-of-pocket and health insurance schemes provide an affordable avenue for them in doing so [51, 52]. Among men, however, we realised it was the richest who had the highest odds of being covered by health insurance, a finding which negates the pro-poor design which underpins the set-up of these schemes. A plausible explanation is the poor execution of healthcare policies such that the rich (who are people capable of affording healthcare costs) prefer paying for healthcare out-of-pocket, which is the main alternative to health insurance [50].

We found that the exposure of both males and females to mass media messages increased the proportion and odds of insurance coverage in urban SSA. This may be attributed to the fact that people in urban areas, who mostly listen to radio will be more encouraged to subscribe to the national health insurance scheme after listening or reading about the benefits and the importance of being covered by health insurance. The observation made in this study points to the importance and the central role that the mass media plays in the dissemination and consumption of health-related knowledge and policies as the media has been recognised as a powerful tool in the dissemination and successful uptake of health interventions [53–57]. A key strength of our study is the use of multi-level analysis which ensured that we accounted for confounding variables effectively. The fact that the DHS is conducted cross-sectionally, however, introduces the possibility of recall bias into the data on the part of the respondents.

## Conclusion

The coverage of health insurance in urban SSA is generally low. This has negative implications for the achievement of universal health coverage by the year 2030. Interventions are, therefore, needed by the respective countries to expand the coverage of health insurance. This can be achieved through increased public education on the benefits of health insurance using the mass media which we found to be an important factor associated with health insurance coverage. The focus of such mass media education could be on the less formally educated urban dwellers, males in the lowest wealth quintile, and young adults (15–29 years).
